# Perilipin 2 modulates adipogenic differentiation of primary dermal fibroblasts and is extracellularly released during adipocyte maturation

**DOI:** 10.1007/s00018-026-06358-z

**Published:** 2026-07-28

**Authors:** Maria Conte, Lorenzo Trofarello, Antonio Chiariello, Gianluca Storci, Paolo Tieri, Giuseppe Conte, Margherita Cortini, Sofia Avnet, Nicola Baldini, Gianandrea Pasquinelli, Sabrina Valente, Stefano Salvioli

**Affiliations:** 1 Department of Medical and Surgical Sciences (DIMEC), University of Bologna, Bologna, Italy; 2 IRCCS Azienda Ospedaliero-Universitaria di Bologna, Bologna, Italy; 3 CNR National Research Council of Italy, IAC Institute for Applied Computing, Rome, Italy; 4 Department of Agriculture, Food and Environment, University of Pisa, Pisa, Italy; 5 Department of Biomedical and Neuromotor Sciences (DIBINEM), University of Bologna, Bologna, Italy

**Keywords:** Perilipin 2, Lipid Droplets, Dermal fibroblasts, Adipogenic differentiation, CD36, Adiposity

## Abstract

Perilipin 2 (PLIN2) is a lipid droplet (LD)-associated protein broadly expressed across tissues, where it regulates fatty acid uptake, lipid storage, and LD dynamics. During adipogenesis, PLIN2 is localized to small LDs in preadipocytes but is absent in mature adipocytes, where it is replaced by other perilipins. Whether PLIN2 loss represents a driver or a consequence of adipogenic differentiation remains unresolved. To address this question, we performed in vitro and ex vivo analyses. Adipogenic differentiation was induced in human primary dermal fibroblasts (DFs), and PLIN2 function was investigated using small interfering RNA (siRNA)-mediated silencing and treatment with Brefeldin A and Monensin (B + M). In parallel, plasma samples from both physically active healthy subjects and patients with mobility impairment were analyzed to assess PLIN2 expression. PLIN2 silencing accelerated adipogenic differentiation, whereas its higher expression induced by B + M markedly delayed this process. Notably, although *Plin2* mRNA became nearly undetectable during differentiation, PLIN2 protein continued to be synthesized and, at variance with DFs, released into the culture supernatant as a circulating form (cPLIN2), partially associated with extracellular vesicles. Consistent with these findings, plasma cPLIN2 levels in vivo positively correlated with increased adiposity and low physical activity. Moreover, skeletal muscle samples from healthy subjects exhibited higher expression of CD36, a recently identified PLIN2 interactor, compared with patients. Collectively, these data identify PLIN2 as a negative regulator of the early phases of adipogenic differentiation and suggest that its secretion may represent a mechanism of interorgan communication potentially mediated by extracellular vesicles.

**Supplementary Information:**

The online version contains supplementary material available at https://doi.org/10.1007/s00018-026-06358-z.

## Introduction

White adipose tissue (WAT) plays a fundamental role not only as the major energy storage but also as an endocrine tissue regulating metabolism, immune response and inflammation through its intense secretory activity. Size and number of adipocytes are highly dynamic and can greatly vary depending on lifestyle habits and physical conditions. Among the conditions that promote adipogenesis, aging is the most universal and unavoidable, thus becoming a risk factor for metabolic disorders, such as obesity, insulin resistance, and dyslipidemia, characterized by a state of chronic inflammation. The main and most prominent feature of WAT is the accumulation of lipids into specialized organelles known as lipid droplets (LDs), a phenomenon observed during adipogenic differentiation. During this process, LDs progressively increase in both size and number that finally fuse together in one single giant LD that occupies most of the mature adipocyte cytoplasm. LD formation and metabolism are strongly driven by the activity of Perilipins (PLINs), a family of five proteins (PLIN1-5) that decorate the membrane of LDs and play a pivotal role in energy storage, metabolism and signal transduction. These proteins are differentially expressed in several tissues: PLIN2 and PLIN3 are the most ubiquitous, PLIN5 is specific for metabolically active tissues, while PLIN1 and PLIN4 are specific for WAT [1]. The pattern and intensity of PLINs expression may vary with age. In fact, it has been reported that PLIN2 intracellular levels increase with age in skeletal muscle and neurons [2–4] and this phenomenon likely represents an attempt to protect cells from lipotoxicity via lipid storage in non-adipose tissues. PLIN2 also plays a role in LD biogenesis and secretion in milk [5, 6]. However, PLIN2 is only present on small LDs of preadipocytes during the early stages of adipogenic differentiation but not in mature adipocytes, where it is replaced by PLIN1, which regulates triacylglycerol storage in LDs. PLIN1 and PLIN2 have distinct features regarding in particular the capacity to bind LDs. It is reported that PLIN1 binds more efficiently LDs with respect to PLIN2 [7]. Beyond their different affinities for LDs, PLIN1 and PLIN2 also exert distinct biological functions. PLIN1 is highly specialized for mature adipocytes and, upon β-adrenergic stimulation, undergoes protein kinase A (PKA)-dependent phosphorylation, promoting the recruitment of CGI-58 and hormone-sensitive lipase (HSL) to regulate triglyceride mobilization and lipolysis [8]. Conversely, PLIN2 mainly participates in the formation and stabilization of nascent LDs and in intracellular lipid storage [9]. Moreover, LD association is a major determinant of PLIN protein stability, as both PLIN1 and PLIN2 undergo rapid degradation when not associated with LDs [10, 11]. Altogether, these observations indicate that the PLIN2-to-PLIN1 transition represents a critical step in adipocyte maturation and LD remodeling rather than simply a switch in perilipin expression.

Whether the loss of PLIN2 is merely a consequence of adipogenic differentiation or contributes to the process remains unclear. To clarify this aspect, we induced adipogenic differentiation in human primary dermal fibroblasts (DFs) in the presence or absence of *Plin2* expression. This model was chosen because DFs normally express only PLIN2 and PLIN3, but not PLIN1 or other PAT family members [12]; therefore, they represent a valuable and simplified model to study the role of PLIN2 in adipogenic differentiation.

PLIN2 also exists as a circulating protein (cPLIN2) in several body fluids besides milk [13], including plasma and urine. In these latter, elevated cPLIN2 levels have been associated with the presence and severity of various disorders, including certain cancers [14, 15]. However, plasma cPLIN2 levels are also detectable in healthy subjects, and are strongly associated with fat mass and inversely with lean mass [16]. Considering that WAT does not express PLIN2, these findings raise questions about the main source and physiological role of cPLIN2. Taking advantage of the same experimental model, we therefore tested the hypothesis that cPLIN2 is secreted by cells undergoing adipogenic differentiation.

## Materials and methods

### Cell culture and treatments

Human primary dermal fibroblasts (DFs), isolated from biopsies of sun-protected areas (abdominal or leg skin) of four healthy donors, were purchased from CTI Biotech, cultured in high-glucose Dulbecco’s Modified Eagle Medium supplemented with 10% heat-inactivated fetal calf serum (FCS), penicillin (100 units/mL), streptomycin (100 mg/mL), and 2 mM L-glutamine (all from Sigma-Aldrich) (DMEM), and maintained in a humidified 5% CO2 incubator at 37 °C. DFs between the 7th and 10th doubling passages were used. In each experiment, after reaching 80% confluence, cells were detached from the flask using 1X Trypsin solution (Sigma-Aldrich) and seeded into 6- or 12-well plates for subsequent treatments.

For adipogenic differentiation experiments, 24 h after seeding, DMEM was replaced with adipogenic differentiation medium (ADM; StemPro^®^ Adipogenesis Differentiation Kit, from Gibco – Thermo Fisher Scientific). DFs were cultured in ADM for 3 to 15 days, then cell pellets and supernatants were collected and stored at -80 °C for subsequent analyses.

Protein Transport Inhibitor Cocktail (containing Brefeldin A and Monensin, eBioscience™ - Thermo Fisher Scientific) was added to fresh medium at the manufacturer’s recommended concentration (typically 2 µL/mL). Cells were treated with the cocktail between the 2nd and the 3rd day of adipogenic differentiation and incubated for 18 h before harvesting. This time window was chosen since at longer incubation times with ADM, *Plin2* gene expression was already reduced, making the treatment ineffective, and, on the other hand, consistently longer treatments would cause stress to cells, therefore results would be difficult to be attributed only to PLIN2 accumulation.

Treatment with oleic acid (OA) was performed by incubating DFs with 200 µM OA in 0.6% bovine serum albumin (BSA) (Sigma-Aldrich) for 3 to 9 days. Incubation with 0.6% BSA alone was used as a negative control.

### Subjects and sampling

Subjects analyzed in this study were recruited in the framework of the EU Project “MYOAGE”. The study protocol was approved by the local ethics committees: Istituto Ortopedico Rizzoli and Sant’Orsola-Malpighi University Hospital (Bologna–Italy) (ethical clearance no. 10823 issued on April 26, 2010). All subjects signed an informed consent before entering the study and ethical aspects for aging research were considered, according to [17].

Subjects were divided into two groups: (i) active healthy subjects (HS) (mean age 57.1 ± 24.87 years); (ii) patients with mobility impairment (MIP) (mean age 60.54 ± 19.44 years). Exclusion criteria for HS included comorbidities, inability to walk 250 m, medication use, recent immobilization (in the last 3 months) and orthopedic surgery (in the last 2 years). For MIP, exclusion criteria comprised major diseases (chronic kidney or liver diseases, severe diabetes, rheumatic, neuromuscular or malignant disorders), infections, chronic steroid use, significant psychiatric conditions, substance abuse, prior hip surgery. Blood samples were collected in the morning after overnight fasting.

Plasma samples from 48 HS and 74 MIP were centrifuged (15 min, 2,000 x g, 4 °C), then frozen at -80 °C. Muscle biopsies of the *Vastus lateralis* were collected from 8 HS and 15 MIP, immediately frozen in liquid nitrogen and stored at -80 °C. Height and weight were measured to calculate BMI (kg/m2). Quadriceps strength (QS) was measured as previously described [2, 3]. Only for HS, a whole-body scan to detect total fat and lean mass was performed. The scan was performed using Dual-energy X-ray absorptiometry (DXA) (Hologic QDR 4500, version 12.4, Hologic Inc., Bedford), by a trained technician.

### In vitro gene expression knockdown

*Plin2* and *Plin3* gene expression downregulation were obtained by RNA interference (RNAi), using predesigned siRNAs (Cohesion Biosciences), according to the silencing protocol as already described [12]. Transfection was performed in 6- or 12-well plates using ScreenFect siRNA reagent (ScreenFect GmbH); after 24 h of incubation with the siRNA (scramble siRNA was used as a control), the medium was replaced with fresh DMEM or ADM. Cell pellets and supernatants were collected after 3, 6, and 9 days of incubation for subsequent analyses.

### RNA extraction and Real-time RT-PCR

Total RNA was extracted from both cells and biopsies using the EasyPure RNA kit (TransGen Biotech Co., Ltd), following the manufacturer’s instructions. RNA quantification was performed using the ScanDrop2 Series spectrophotometer (Analytik Jena).

For cDNA synthesis, HIScript III RT SuperMix for qPCR (+ gDNA wiper) kit was used (Vazyme Biotech), according to the manufacturer’s instructions.

Gene expression was analyzed by Real-time RT-PCR using iTaq Universal SYBR Green (Bio-Rad) on a Rotor Gene Q 6000 (Qiagen). A comparative analysis was performed using *Gapdh* as reference gene, and relative expression was calculated by the 2^−ΔΔCt^ method. Validated primers were purchased from Bio-Rad (www.bio-rad.com/PrimePCR). All experiments were performed at least three times.

### Protein extraction and Western blotting analysis

Protein extraction was performed using RIPA buffer containing 50 mM Tris HCl (pH 8), 150 mM NaCl, 0.5% Sodium deoxycholate, 0.1% SDS and 1% Triton X-100. Protease and phosphatase inhibitors (Sigma-Aldrich) were added to the lysis buffer immediately before use. Cell pellets were resuspended in the lysis buffer by pipetting, incubated on ice for 15 min with intermittent vortexing and then centrifuged at 15,000 rpm for 15 min at 4 °C. Supernatants were collected,and protein concentration was determined using the Bradford assay. Purified proteins were then stored at -80 °C until use. Protein expression was analyzed by Western blotting. Equal amounts of protein (20 µg) were separated on 4–15% Mini-PROTEAN^®^ TGX™ Precast Protein Gels (Bio-Rad) and transferred onto a nitrocellulose membrane using a Trans-Blot system (Bio-Rad) and then incubated with the following primary antibodies: PLIN1 (rabbit monoclonal, Cell Signaling Technology, D1D8, 1:1000), PLIN2 (rabbit polyclonal, LS Bio, LS-B4850, 1:500), PLIN3 (rabbit polyclonal, LS Bio, LS-B2539, 1:2000). GAPDH (mouse monoclonal, Cohesion Biosciences, CPA9067, 1:20000) was used as a loading control. Signals were developed using an enhanced chemiluminescence (ECL) substrate (Agrisera), and immunoreactive bands were detected using a ChemiDoc™ Imaging System (Bio-Rad).

### Enzyme-linked immunosorbent assay (ELISA)

LEPTIN, cPLIN2 and RESISTIN levels were measured by ELISA using commercially available kits (Human LEPTIN Quantikine, DLP00 and Human RESISTIN Quantikine DRSN00, both R&D Systems; Human ADRP, E-EL-H0278, Elabscience) according to the manufacturers’ instructions. The Human ADRP/PLIN2 ELISA Kit employs a sandwich ELISA format based on two PLIN2-specific antibodies and is validated by the manufacturer for quantitative detection of human PLIN2 (ADRP/adipophilin) in biological samples. Standard curves were generated from serial dilutions of the provided standards. Cell culture supernatants were assayed undiluted, whereas plasma for cPLIN2 determination was diluted 1:25 before analysis. All samples were measured in duplicate in at least two independent assays. Absorbance was read using a Glomax^®^ Discover microplate reader (TM397, Promega), and concentrations were calculated from the corresponding standard curves according to the manufacturers’ recommendations.

### Holotomographic microscopy

LDs content was analyzed using a Nanolive CX-F holotomography microscope (Tolochenaz, Switzerland) equipped with a 60X objective. Cells were seeded on glass-bottom dishes (Ibidi, Gräfelfing, Germany). Live, label-free imaging was performed 3 and 9 days after adipogenic differentiation. A 24-hour time-lapse live imaging was also performed between days 2 and 3 of differentiation. Lateral (x-y) and axial (z) resolutions were 200 nm and 400 nm, respectively. Auto-calibration accounted for the immersion medium (DMEM, RI: 1.3370). LD quantification and metrics (LD area and dry mass) were automatically obtained with Eve Analytics software (Nanolive).

### Lipid staining and analysis

Lipid staining was performed with Oil Red O. After 14 days, cells were fixed in formalin (10 min, RT), rinsed, and stained with Oil Red O (15 min under agitation). Images were acquired by phase-contrast microscopy. For quantification, Oil Red O was eluted with isopropanol (15 min, RT, in the dark) and absorbance was measured at 500 nm using a Multiskan SkyHigh microplate reader (Thermo Fisher Scientific).

### Transmission and scanning electron microscopy (TEM, SEM)

Ultrastructural morphology was investigated using electron microscopy. For TEM, cells were seeded in 12-well plates at a density of 31’250 cells/well and induced to adipogenic differentiation for 14 days; control cells were maintained in DMEM. Then, cellular samples were fixed in 2.5% buffered glutaraldehyde (20 min, RT) and centrifuged. Cell pellets were stored at 4 °C. Pellets were post-fixed in 1% osmium tetroxide for 1 h and an ethanol dehydration with increased concentrations at RT was then performed. Ultrathin sections were collected on grids, stained with uranyl acetate and lead citrate and examined using a CM 10 Transmission Electron Microscope (FEI, Thermo Fisher Scientific) equipped with a camera for acquiring digital images using proprietary software. For SEM, cells were plated on glass coverslips at a density of 31’250 cells/well and cultured in DMEM and ADM for 9 days. After washing with PBS, cells were fixed in 2.5% buffered glutaraldehyde overnight at 4 °C. Cells were post-fixed in 1% osmium tetroxide, rinsed in water and dehydrated with different ethanol concentrations (70%, 95%, 100%, RT, 15 min). Cells were dried in a 1:1 solution of absolute ethanol and hexamethyldisilazane (HMDS, Merck) followed by pure HMDS and finally air-dried; each passage was performed at RT for 30 min. Samples were mounted on aluminum stub supports (Multilab type stub pin ½, Surrey, UK) and coated with gold in a S150A Sputter Coater before observation at 10 kV with a SEM microscope (Thermo Scientific Quattro S) connected with a digital camera.

### Extracellular vesicles (EVs) analysis

EVs phenotype analysis was performed with the ExoView^®^ R200 instrument (Unchained Labs). Cell culture supernatants were centrifuged at 5000 x g for 15 min to remove cellular debris and then spotted onto an ExoView chip with 1:2 dilution in incubation buffer, following the manufacturer’s instruction. The signals from DMEM and ADM alone were used as negative controls for background correction. Detection antibody employed was Perilipin-2/ADFP-Alexa Fluor^®^ 488 Antibody (NB110-40877AF488, Novus Biologicals).

To further assess PLIN2 expression in vesicles, EVs were isolated from supernatants (500 µl) of cells cultured in either DMEM or ADM for 9 days using Immunobeads for Overall Exosome Capture (HansaBioMed Life Sciences), according to the manufacturer’s protocol. Equal volumes of EVs suspensions were then spotted onto a pre-wetted nitrocellulose membrane using a Bio-Dot apparatus (Bio-Rad). Membranes were then blocked in 5% (w/v) non-fat dry milk in Tris-buffered saline containing 0.1% Tween-20 (TBS-T) for 1 h at room temperature. After blocking, membranes were incubated overnight at 4 °C with PLIN2 primary antibody (1:500) diluted in blocking buffer; primary antibody against CD63 (mouse monoclonal, Invitrogen, Ts63, 1:500) was used as apositive control. Following incubation, membranes were washed three times with TBS-T and incubated with appropriate HRP-conjugated secondary antibodies for 1 h at 4 °C. After extensive washing, immunoreactive signals were detected using an enhanced chemiluminescence (ECL) substrate (Agrisera) and imaged using a ChemiDoc™ Imaging System (Bio-Rad).

### Membrane integrity test

Plasma membrane integrity was assessed by flow cytometry analysis upon Propidium Iodide (PI) staining. Briefly, cells cultured in DMEM or ADM for 9 days were harvested, washed with phosphate-buffered saline (PBS), and incubated with PI (200 µg/mL in PBS) for 5 min at room temperature, protected from light. Samples were immediately analyzed using a CytoFLEX S flow cytometer (Beckman Coulter). Appropriate unstained and PI-positive controls were used to set the gates, and debris and doublets were excluded. PI-positive cells, indicative of compromised plasma membrane integrity, were quantified as a percentage of total cells.

### Identification of predicted protein-protein interactions

Under the hypothesis that cPLIN2 can interact with membrane proteins even in the absence of experimentally validated evidence, such interactions may be inferred from structural compatibility rather than prior empirical characterization. We propose that cPLIN2 is eventually taken up by specific cells or tissues, where it may engage in previously uncharacterized interactions with membrane-associated proteins. Following this hypothesis, predicted protein-protein interactions (PPIs) with PLIN2 were systematically searched using two complementary computational databases, IID and PrePPI.

The Integrated Interactions Database IID (https://iid.ophid.utoronto.ca) [18] integrates and stores high-confidence PPIs predicted by state-of-the-art computational methods, as described in several studies [19–22]. The search for PLIN2 interactors was carried out filtering with the following criteria: (1) query IDs: PLIN2; species where IDs are from: human; retrieval strategy: retrieve all PPIs of query proteins; (2) Filter interactions by evidence – specific PPI sources: predicted.

Additionally, the PREdicted Protein-Protein Interactions database PrePPI (https://honiglab.c2b2.columbia.edu/PrePPI/) [23] was employed, which utilizes a Bayesian framework to compute likelihood ratios (LR) for protein pairs based on multiple lines of evidence: structural modeling scores indicating structural similarity, protein-peptide interactions, phylogenetic profiles, RNA expression profiles, and Gene Ontology annotations. In PrePPI, the identification of potential interactors for a given gene is performed by entering the gene of interest (here PLIN2), which returns a results table ranked by the likelihood ratios of the predicted interactions.

### Statistical analysis

The data are shown as mean ± standard deviation (SD) for in vitro experiments and as mean ± standard error (SE) for human cohort data, as specified in the corresponding figure legends. The Shapiro-Wilk normality test was performed to check whether the data were normally distributed. Accordingly, comparisons between control and differentiated cells for real time RT-PCR and Oil Red O experiments were performed using Student’s *t*-test. For all other analyses, the Mann-Whitney *U* test was applied to compare two independent groups, while the Kruskal-Wallis test was used to determine differences between more than two independent groups. Bonferroni-corrected pairwise comparisons were also applied. To evaluate whether the association between study groups (HS *versus* MIP) and plasma cPLIN2 was independent of potential confounding factors, a multiple linear regression analysis was performed with plasma cPLIN2 as the dependent variable and study group, sex, age, BMI, LEPTIN, and RESISTIN as independent variables. Regression assumptions, including multicollinearity, were assessed before model interpretation. *P* values < 0.05 were considered statistically significant. Statistical analyses were performed using SPSS Statistics version 28.0.0.0 (SPSS Inc., Chicago, IL, USA), except for the multiple linear regression analysis, which was performed using R (version 4.4.2; R Foundation for Statistical Computing, Vienna, Austria).

## Results

### The expression pattern of Perilipins changes during adipogenic differentiation of human DFs

**Fig. 1:**
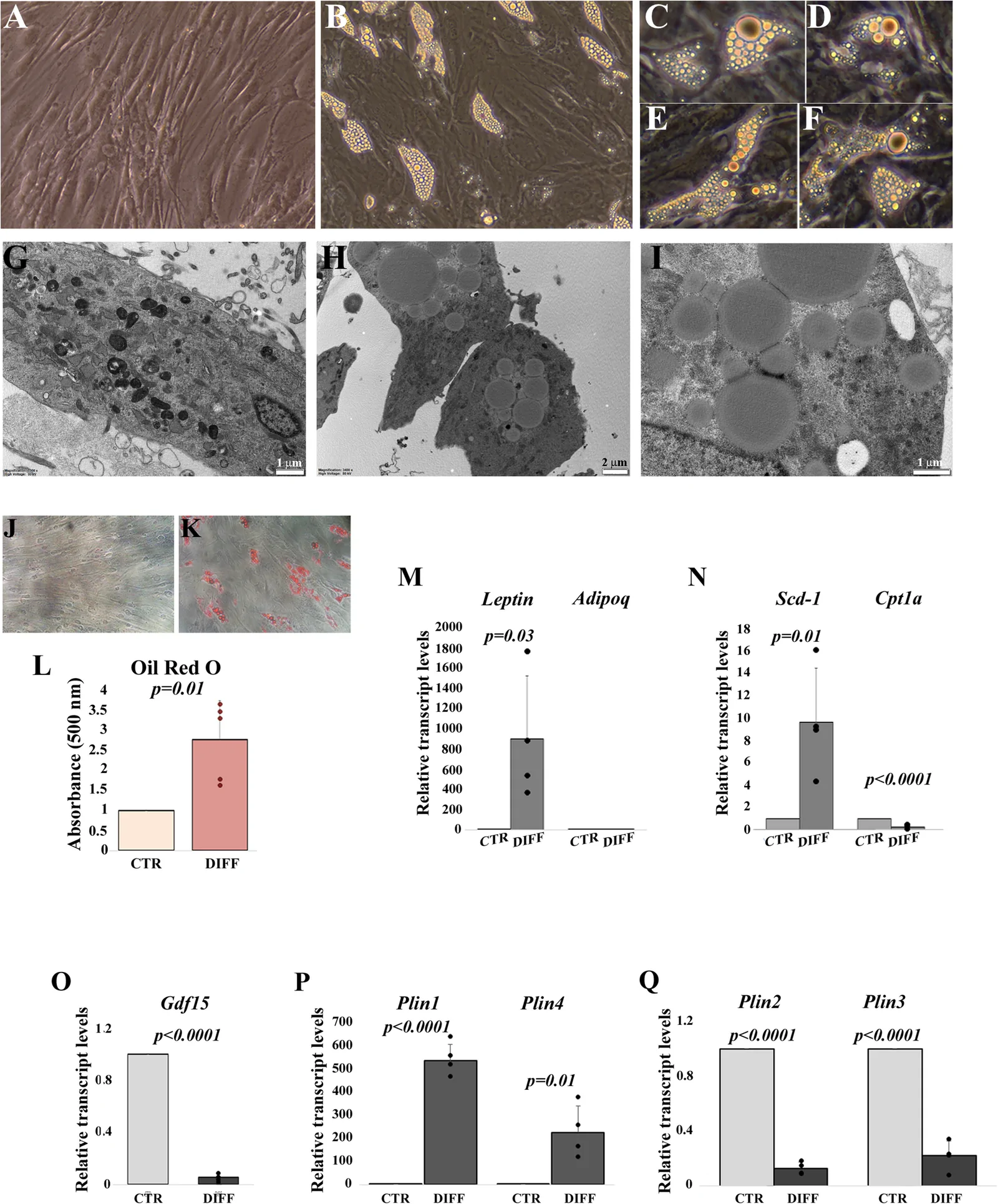
Adipogenic differentiation of dermal fibroblasts** (DFs).** (**A-F**) Representative light microscopy images of DFs cultured in DMEM (control, CTR; **A**), or adipogenic differentiation medium (ADM) (differentiated cells, DIFF; **B-F**), showing an accumulation of lipid droplets in DIFF cells; magnification 40X. (**G-I**) Representative transmission electron microscopy images of CTR (**G**) and DIFF cells (**H-I**); scale bars: 1 μm (**G**), 2 μm (**H**), 1 μm (**I**). (**J-K**) Oil Red O staining of CTR (**J**) or DIFF cells (**K**); magnification 20X. (**L**) Quantification of lipid content in CTR and DIFF cells; staining was performed on four independent DFs preparations. (**M-Q**) Real-time RT-PCR analysis of gene expression in CTR and DIFF cells. Oil Red O staining and real-time RT-PCR analyses were performed on five and four independent DF lines, respectively; all experiments were performed in technical triplicate for each biological replicate, and the mean values were used for statistical analysis. Data are expressed as mean ± SD. Statistical significance was assessed using Student’s t-test

Human primary DFs cultured up to 15 days in adipogenic differentiation medium (ADM), as expected, displayed an accumulation of intracellular LDs compared to counterparts cultured in DMEM, as observed by both light microscopy (Fig. 1A-F) and TEM analysis (Fig. 1G-I). Accordingly, Oil Red O staining confirmed a significant increase in lipid content (*p* < 0.01), suggesting that DFs were converting into adipocytes (Fig. 1J-L). Adipogenic differentiation was further confirmed by real time RT-PCR analysis of genes involved in fat storage and energy metabolism. After 15 days of culture in ADM, cells expressed the adipocyte-specific gene *Leptin*, but not yet *Adiponectin* (*Adipoq*) (Fig. 1M). Furthermore, the transcript levels of *Stearoyl-CoA desaturase-1* (*Scd-1*), whose expression and activity are essential for WAT, were significantly upregulated in DFs cultured in ADM, as compared to control ones (*p* = 0.01) (Fig. 1N); conversely, the expression levels of *Carnitine palmitoyltransferase 1* (*Cpt-1a*), whose expression is very low in WAT [24], were strongly decreased in DFs in ADM (Fig. 1N). In line with these observations, a decreased expression of *Gdf15*, recently proposed as an inhibitory factor of the early phase of adipogenic differentiation [25], was also observed (Fig. 1O). As previously reported, *Plin2* and *Plin3* are the only two perilipins expressed in human DFs [12], while *Plin1* and *Plin4* are predominantly expressed in adipocytes [1]. Accordingly, upon culture in ADM, the expression levels of *Plin1* and *Plin4* increased, while those of *Plin2* and *Plin3* were decreased (Fig. 1P-Q).

### Adipocyte related genes and LD formation are modulated by PLIN2 during adipogenic differentiation

As previously reported by Brasaemle and colleagues, *Plin2* is expressed in the early stages of adipogenic differentiation and is progressively replaced by *Plin1* during adipocyte maturation [9]. To assess when this transition occurs during DF differentiation, we examined *Plin2* and *Plin1* transcripts by real-time RT-PCR at different time points of adipogenic differentiation. Although LDs became visible by light microscopy only starting from day 6 (Suppl. Figure 1A-D), *Plin2* and *Plin3* transcript levels have already decreased by approximately 70% at day 3 in ADM and continued to decline until becoming almost undetectable at day 15 (Suppl. Figure 1E, F). In contrast, *Plin1* expression was already significantly induced from day 3 and continued to increase strongly at subsequent time points (Suppl. Figure 1G). These results were only partially reflected by Western blotting analysis, in particular PLIN1 protein was not yet detectable by day 9, while PLIN2 remained detectable (Suppl. Figure 1H), confirming previous results [9]. *Leptin* gene transcription was already significantly elevated by day 3, and the corresponding secreted protein was proportionally detectable in the supernatants (Suppl. Figure 1I-J).

We then asked whether the decrease in *Plin2* expression was simply a consequence of adipogenic differentiation or a prerequisite of this process. To test this hypothesis, *Plin2* was silenced using specific siRNA (siPLIN2), which efficiently reduced *Plin2* expression in both control and differentiating DFs (Suppl. Figure 2A). siPLIN2 did not affect *Plin3* in DMEM but increased its expression in ADM (Suppl. Figure 2B), suggesting a compensatory response to *Plin2* silencing. Conversely, siPLIN3 had no effect on *Plin2* expression (Suppl. Figure 2C-D). Although *Plin2* silencing has been reported to induce cell senescence [12], and senescent cells are characterized by LDs accumulation [26], siPLIN2 did not alter*p21* or *Gdf15* expression, markers of senescence and SASP, during differentiation in ADM (Suppl. Figure 2E-F).

**Fig. 2:**
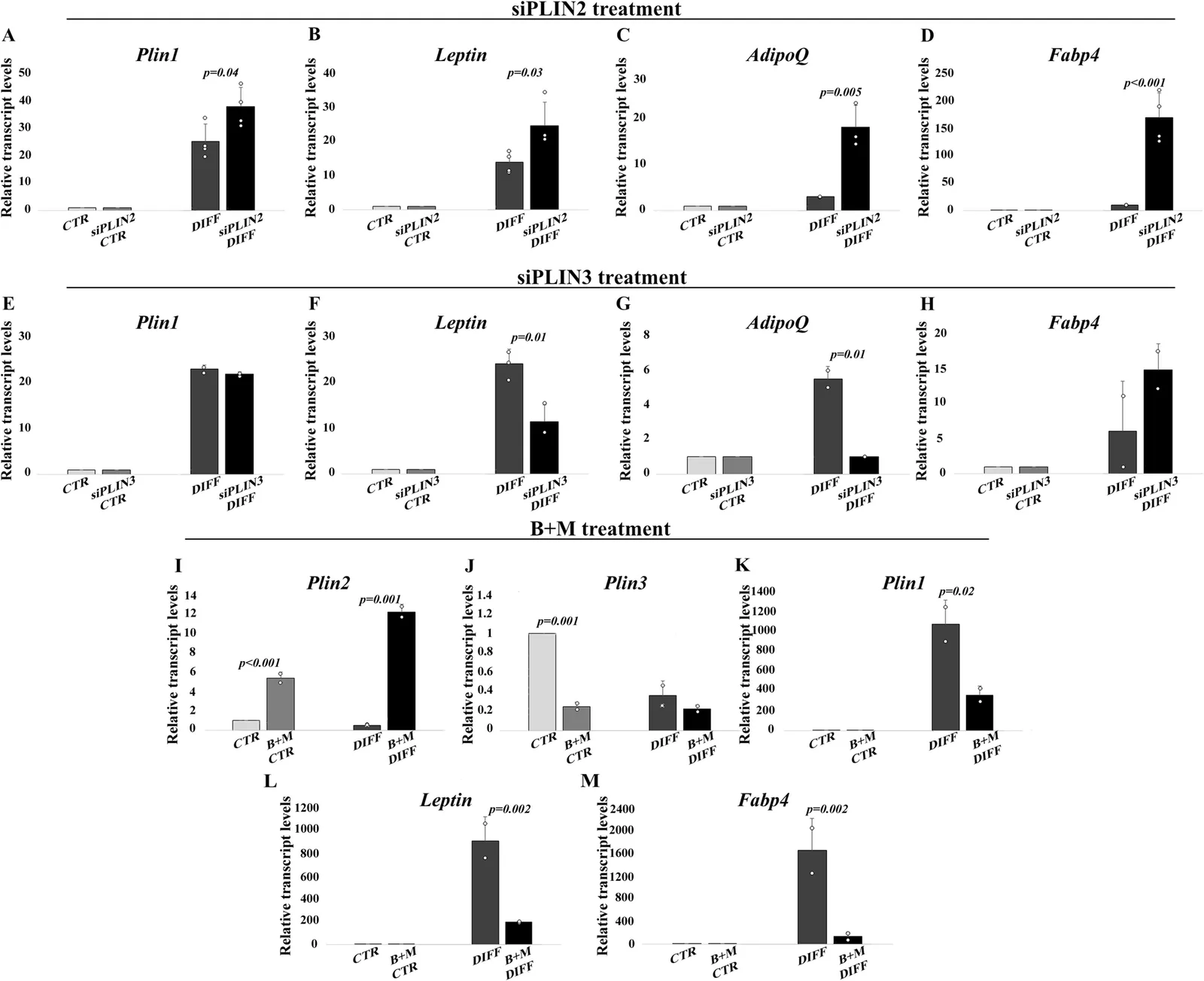
Real-time RT-PCR analysis following *Plin2* or *Plin3* downregulation (siPLIN2 or siPLIN3) or Brefeldin A and Monensin (B + M) treatment. (**A-D**) *Plin1*, *Leptin*, *AdipoQ*, and *Fabp4* transcript levels in DFs cultured in DMEM with or without siPLIN2 (CTR and siPLIN2 CTR), or in adipogenic differentiation medium (ADM) with or without siPLIN2 (DIFF and siPLIN2 DIFF). (**E-H**) *Plin1*, *Leptin*, *AdipoQ*, and *Fabp4* transcript levels in DFs cultured in DMEM with or without siPLIN3 (CTR and siPLIN3 CTR), or in ADM with or without siPLIN3 (DIFF and siPLIN3 DIFF). (**I-M**) *Plin2*, *Plin3*, *Plin1*, *Leptin*, and *Fabp4* transcript levels in DFs cultured in DMEM with or without B + M (CTR and B + M CTR), or in ADM, with or without B + M (DIFF and B + M DIFF). Experiments were performed using four independent DF lines. Real-time RT-PCR analyses were performed in technical triplicate for each biological replicate, and the mean values were used for statistical analysis. Data are expressed as mean ± SD. Statistical significance was assessed using Student’s *t*-test.

Interestingly, the expression of several adipose tissue markers, including *Plin1*, *Leptin*, *Adipoq* and *Fabp4*, was significantly increased following siPLIN2 treatment in ADM compared with ADM alone (Fig. 2A-D). In contrast, this effect was not observed upon siPLIN3 treatment, as the expression of adipocyte-specific genes was comparable to, or lower than, that observed in DFs cultured in ADM alone (Fig. 2E-H). Taken together, these data suggest that the decrease in *Plin2* expression is not merely a consequence of the adipogenic differentiation but may actively facilitate the differentiation process.

To further test this hypothesis, cells were treated with Brefeldin A and Monensin (B + M). In addition to their roles in protein trafficking and lipid distribution and mobilization [27], these compounds have been reported to promote LDs formation, triacylglycerol accumulation and PLIN expression [28–31]. Consistent with this, B + M treatment increased *Plin2* expression in both DMEM and ADM culture conditions (Fig. 2I). By contrast, *Plin3* expression was reduced under the same conditions (Fig. 2J). Accordingly, increased *Plin2* expression was accompanied by reduced expression of the adipogenic markers *Plin1*, *Leptin* and *Fabp4* in DFs cultured in ADM (Fig. 2K-M), while *Adipoq* expression was undetectable (data not shown). These observations further supported the idea that a decreased Plin2 expression may be part of a specific program required for early adipogenic differentiation.

To further investigate whether the effects of B + M on adipogenic differentiation were actually linked to *Plin2* expression, DFs cultured in ADM were subjected to *Plin2* silencing and subsequently treated with B + M. siPLIN2 treatment strongly blunted the rise of *Plin2* expression caused by B + M; nevertheless, the presence of B + M still maintained a slight but significantly higher level of *Plin2* expression, as compared to ADM alone (Suppl. Figure 3 A). Consistently, the expression of *Plin1* was slightly but significantly higher in siPLIN2-B + M as compared to B + M alone (Suppl. Figure 3B). Furthermore, we evaluated *Plin3* expression to determine whether the effects of B + M reflected a broader upregulation of PAT family members. Notably, *Plin3* expression resulted actually decreased upon both B + M and siPLIN2-B + M treatments, suggesting that (i) B + M treatment has different effects on different PAT family members; (ii) B + M-mediated decrease in *Plin3* expression is not rescued by siPLIN2 (Suppl. Figure 3 C). This observation supports the hypothesis that B + M-induced modulation of DFs adipogenic differentiation is primarily associated with changes in *Plin2* expression, which in turn contributes to the attenuation of *Plin1* expression. Overall, these additional data further strengthen the association between *Plin2* expression and the slowing of adipogenic differentiation in this model.

**Fig. 3:**
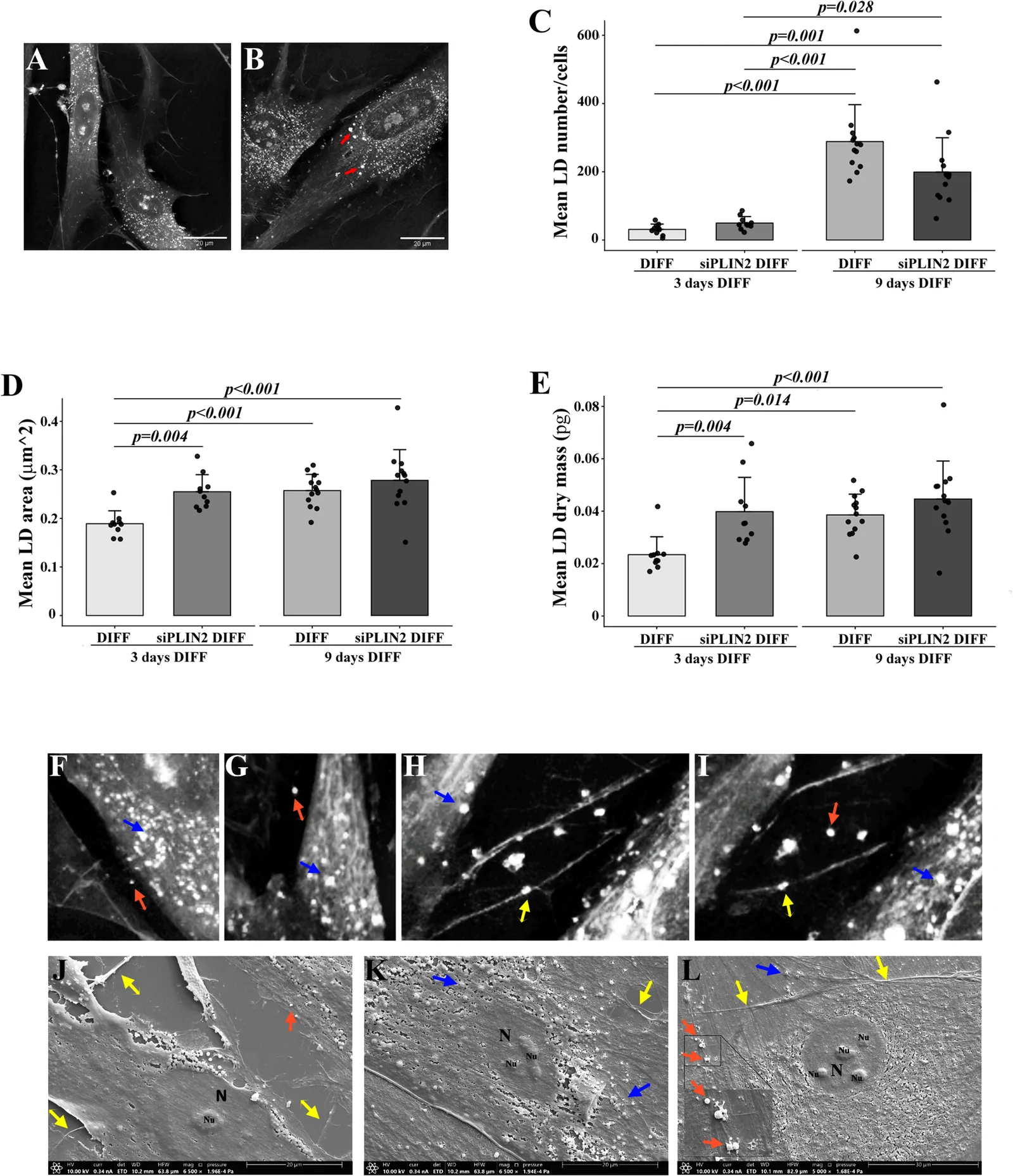
Lipid droplets (LDs) analysis in dermal fibroblasts (DFs) during adipogenic differentiation. (**A-B**) Representative Nanolive images of DFs cultured for 3 days in adipogenic differentiation medium (ADM) with (**A**) or without (**B**) siPLIN2; larger LDs are visible in siPLIN2-treated cells (red arrows). (**C-E**) Quantitative analysis of LD morphometric parameters after 3 and 9 days of culture in ADM with or without siPLIN2 (DIFF and siPLIN2 DIFF): LD number per cell (**C**), LD area (**D**), and LD dry mass (**E**), normalized to the number of cells in each field of view. Nanolive analyses were performed on one DF line and repeated in two independent experiments. Quantification was obtained from 24-h time-lapse acquisitions, with images acquired every 2 min. The numbers of analyzed cells were 10 at day 3 and 13 at day 9, for both conditions (DIFF and siPLIN2 DIFF). (**F-I**) Representative 24-h Nanolive time-lapse live-cell images of DFs after 3 days of culture in ADM, showing intracellular LDs (blue arrows), extracellular LDs (red arrows), and LDs within tunneling nanotubes (yellow arrows). (**J-L**) Representative scanning electron microscopy images of DFs after 3 days in ADM, showing intracellular LDs (blue arrows), extracellular LDs (orange arrows), and LDs within tunneling nanotubes (yellow arrows). N, nucleus; n, nucleolus. Data are expressed as mean ± SD. Statistical analysis was performed using the Kruskal-Wallis test followed by Bonferroni-corrected pairwise comparisons

Since adipogenic differentiation is accompanied by a significant rise in LDs number and size, we assessed the impact of *Plin2* downregulation on these parameters. Live, label-free imaging was performed using Nanolive 3D holotomography microscope platform, which enables LDs identification based on their high refractive index. LDs analysis was carried out after 3 and 9 days of culture in ADM, in presence or absence of siPLIN2. After 3 days of culture in ADM, LDs from siPLIN2-treated cells exhibited larger area and dry mass but not number compared to control (Fig. 3A-E). No differences were observed between groups at day 9. Notably, at day 9 these morphometric parameters were comparable to those measured at day 3 following siPLIN2 treatment, suggesting that PLIN2 plays a role in modulating LDs growth during the early stages of adipocyte maturation. Additionally, at day 9 the number of LDs tended to be lower while LD area and mass tended to be higher in siPLIN2-treated cells, thus suggesting a more pronounced phenomenon of LD fusion. In addition, 24-h Nanolive time-lapse live imaging revealed the presence of LDs not only within the cytoplasm but also inside cytoplasmic prolongation and in the extracellular space (Fig. 3F-I). The presence of extracellular LDs was further confirmed by SEM, which revealed rounded particles outside the cells (Fig. 3J-L). Moreover, SEM analysis showed a diffuse communication network formed by long, thin cytoplasmic protrusions consistent with tunneling nanotubes (Fig. 3J-L), as previously described [32].

### PLIN2 is extracellularly released during adipogenic differentiation

**Fig. 4:**
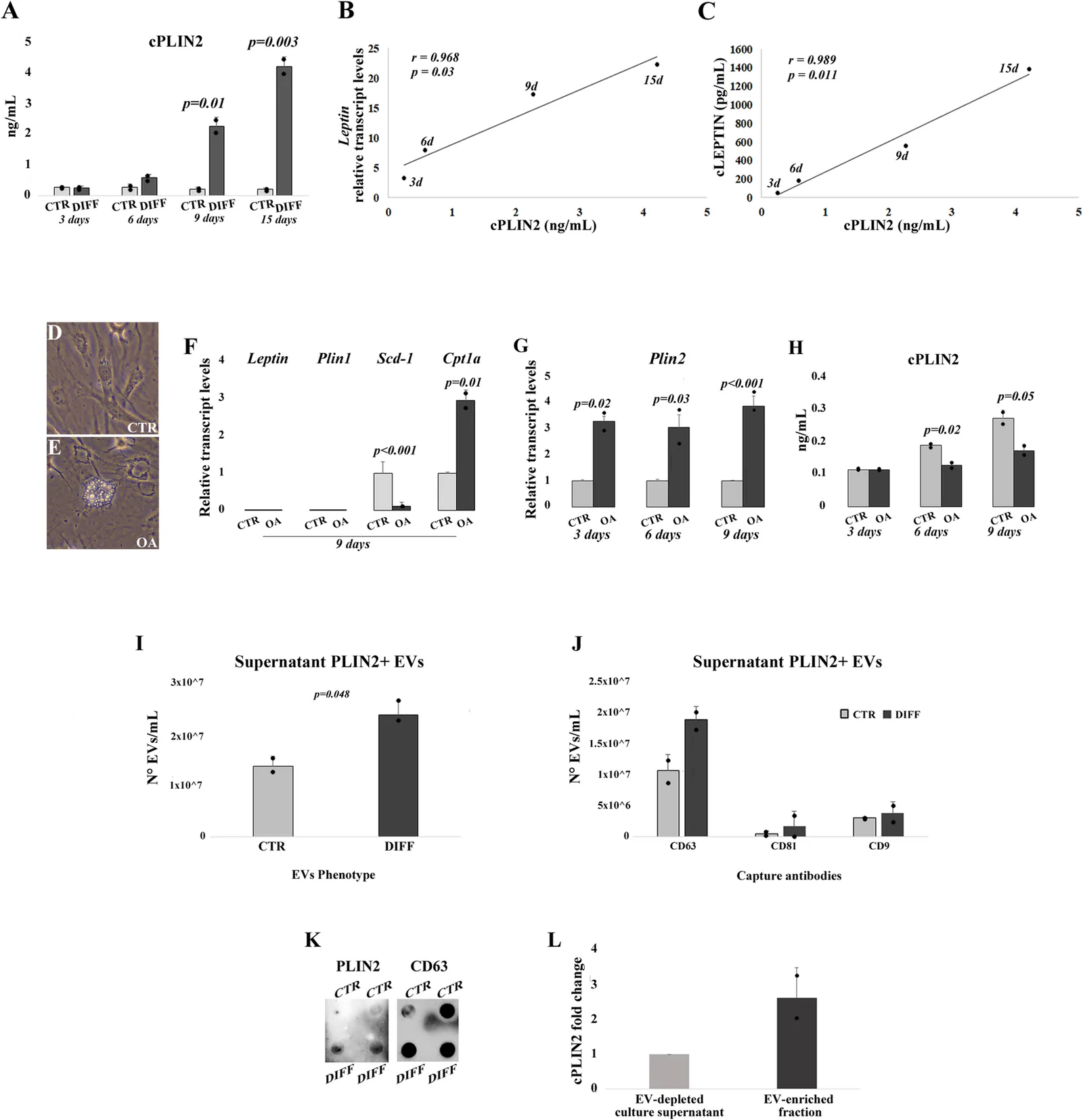
Circulating PLIN2 (cPLIN2) analysis in dermal fibroblasts (DFs) culture supernatants. (**A**) ELISA quantification of cPLIN2 in supernatants from DFs cultured in DMEM (control, CTR) or in adipogenic differentiation medium (ADM, differentiated cells, DIFF), collected at days 3, 6, 9, and 15. (**B-C**) Linear regression analysis of cPLIN2 with *Leptin* transcript levels (**B**) and secreted LEPTIN (cLEPTIN) (**C**) evaluated at days 3, 6, 9, and 15 of culture in ADM. Pearson correlation coefficient (r) and p-value are shown. Analyses were performed using four independent DF lines (biological replicates). ELISA and real-time RT-PCR measurements were performed in technical triplicate, and the experiment was repeated at least five independent times. (**D-E**) Representative light microscopy images of DFs cultured in DMEM alone (CTR, **D)** or supplemented with oleic acid (OA, **E**). (**F**) Real-time RT-PCR analysis of *Leptin*, *Plin1*, *Scd-1*, and *Cpt1A* transcripts in DFs after 9 days of culture in DMEM with or without OA. (**G-H**) Real-time RT-PCR analysis of *Plin2* transcript levels (**G**) and ELISA quantification of cPLIN2 (**H**) in DFs cultured in DMEM (CTR) or in ADM (DIFF), at days 3, 6, and 9. Analyses were performed using two independent DF lines and repeated in two independent experiments. Real-time RT-PCR and ELISA measurements were performed in technical triplicate. (**I**) ExoView analysis of PLIN2^+^ extracellular vesicles (EVs) in supernatants from DFs cultured in DMEM (CTR) or ADM (DIFF) for 9 days. (**J**) Analysis of tetraspanin expression associated with PLIN2^+^ EVs present in CTR and DIFF supernatant. (**K**) Dot blot analysis of PLIN2 and CD63 in EV fractions isolated from CTR and DIFF DF supernatants. (**L**) cPLIN2 levels in EV-depleted supernatants and EV-enriched fractions. ExoView analyses were performed using two independent DF lines and repeated in two independent experiments. Each sample was analyzed in technical duplicate. Statistical significance was assessed using Student’s *t*-test for real-time RT-PCR and ELISA analyses, and Mann-Whitney *U* test for EVs analyses

We previously reported that in vivo circulating PLIN2 (cPLIN2) levels are strongly associated with body fat mass and plasma LEPTIN levels; accordingly, we hypothesized that cPLIN2 could serve as an indicator of body adiposity [16]. However, the origin of such cPLIN2 is not yet clear. As described above, despite a dramatic decrease in *Plin2* mRNA levels, by day 9 of differentiation the levels of PLIN2 protein appeared still detectable. In order to test the hypothesis that cPLIN2 is released by cells under adipocytic differentiation, we investigated whether cPLIN2 could be detected in the supernatant of DFs cultured in either DMEM or ADM by ELISA. cPLIN2 levels resulted markedly increased in the supernatant of DFs cultured in ADM compared with those cultured in DMEM (Fig. 4A). Consistent with our previous findings [16], cPLIN2 levels strongly correlated with both *Leptin* transcript and secreted protein (cLEPTIN) levels (Fig. 4B-C). Collectively, these results suggest that, despite a strong downregulation of *Plin2* transcription, PLIN2 protein is still present and extruded by cells undergoing adipogenic differentiation.

The mechanisms of extrusion of cPLIN2 are not clear, however, we can exclude that the rise in cPLIN2 levels is due to a passive leakage resulting from membrane damage. DFs in DMEM or ADM did not show gross morphological alterations by day 9 (Suppl. Figure 4A, B), and plasma membrane integrity test (assessed by flow cytometry analysis upon PI staining) showed no major differences between DFs under DMEM or ADM conditions (Suppl. Figure 4C, D).

To test whether cPLIN2 release is actually due to adipogenic differentiation or more simply to lipid accumulation, we treated DFs with oleic acid (OA) in DMEM. After 9 days of OA treatment, LDs accumulation was observed in DFs (Fig. 4D-E). However, in contrast to what was observed during culture in ADM (Suppl. Figure 1E), upon OA treatment *Plin1* and *Leptin* transcripts were undetectable, while those of *Scd-1* and *Cpt1a* were decreased and increased, respectively (Fig. 4F), i.e., the opposite of what was observed after adipogenic differentiation (Fig. 1N), and more importantly, *Plin2* gene expression was increased but not cPLIN2 levels (Fig. 4G-H). These data confirm that cPLIN2 secretion occurs specifically during adipogenic differentiation.

We then wondered whether the appearance of cPLIN2 could be linked to LD release. Several studies have shown that adipocytes secrete large numbers of EVs, which contribute to inter-organ communication [33]. Furthermore, Genard and colleagues recently demonstrated that LDs influence EV biogenesis and content, and that LDs and EVs share several proteins [34]. Therefore, to test whether the increase in cPLIN2 could be attributed to EVs production and release, we analyzed the supernatant of DFs cultured in either DMEM or ADM using refractometric ExoView analysis. PLIN2 was detected on EVs. Notably, after 9 days of culture in ADM, PLIN2^+^ EVs were significantly increased in the supernatant (*p* < 0.05) (Fig. 4I-J). To further substantiate this result, a dot blot analysis was performed indicating that supernatants from cultures in ADM contained more PLIN2^+^ CD63^+^ EVs with respect to control culture supernatants (Fig. 4K). Finally, we separated EV-enriched and EV-depleted fractions from the same ADM supernatant and observed a consistent fold increase in cPLIN2 content, as assessed by ELISA (Fig. 4L). These findings indicate that cPLIN2 is at least partially associated with EVs. Nonetheless, other forms of cPLIN2 (e.g. present either in a soluble form or in complex with binding proteins) cannot be ruled out and warrant further investigation.

### Elevated in vivo plasma levels of cPLIN2 are associated with muscle inactivity

**Fig. 5:**
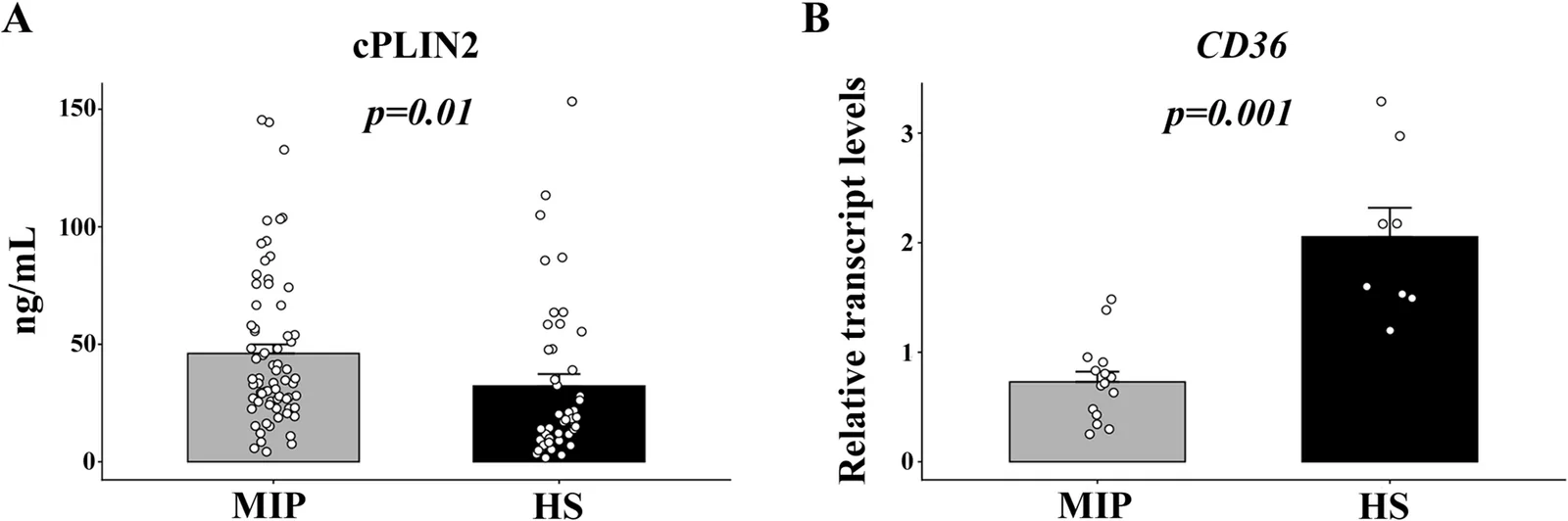
cPLIN2 and *CD36* expression in muscle samples from mobility impairment patients (MIP) and healthy subjects (HS). (**A**) Circulating PLIN2 (cPLIN2) levels measured by ELISA in 48 HS and 74 MIP. (**B**) Relative *CD36* transcript levels measured in *V. lateralis* biopsies from 8 HS and 15 MIP. Data are expressed as mean ± SE. Statistical significance was determined using the Mann-Whitney U test

Previous studies have reported that cPLIN2 is associated with BMI, metabolic and inflammatory markers and elevated in critically ill and cancer patients [14–16, 35, 36]. However, its physiological role remains largely unclear. Given the previously reported inverse association between plasma cPLIN2 and lean body mass [16], we hypothesized that cPLIN2 may mediate lipid transport or signaling between adipose tissue and peripheral organs, particularly skeletal muscle (SM). To explore this hypothesis, we analyzed cPLIN2 levels in a previously described cohort [3], including healthy subjects (HS) and patients with mobility impairment (MIP), characterized by low levels of muscle mass and strength due to prolonged inactivity. Consistent with our previous results, plasma cPLIN2 levels were positively correlated with LEPTIN plasma levels and BMI in the overall cohort. In HS, where also body composition analyses were available, cPLIN2 also showed a positive correlation with fat mass, and negative with lean mass (Suppl. Table 1). Notably, cPLIN2 levels were negatively associated with quadriceps strength normalized for BMI in all subjects from both groups (Suppl. Table 1). Moreover, plasma cPLIN2 levels, normalized for BMI and LEPTIN (considered as a proxy of adiposity), were higher in MIP than in HS (Fig. 5A). To evaluate whether this difference was independent of potential demographic and metabolic confounders, we performed a multiple linear regression analysis including study group (HS *versus* MIP), sex, age, BMI, LEPTIN and RESISTIN as independent variables (Suppl. Table 2). After adjustment, study group remained independently associated with plasma cPLIN2 levels (β = −7.70, 95% CI − 14.43 to − 0.83, *p* = 0.029), whereas LEPTIN was the only other significant independent predictor (β = 2.43, 95% CI 2.00–2.87, *p* < 0.001). Age, sex, BMI and RESISTIN were not significantly associated with plasma cPLIN2. These findings indicate that the higher circulating cPLIN2 levels observed in MIP are not solely explained by differences in the analyzed demographic and metabolic variables, supporting an association between cPLIN2 and impaired muscle status.

To lend support to this hypothesis, we searched for possible cPLIN2 interactors expressed in SM. We therefore performed a complementary computational PPI prediction analysis using the IID and PrePPI databases. This analysis identified 20 putative PLIN2 interactors in IID and 28 predicted interactors in PrePPI (Suppl. Tables 3 and 4). Three candidate interactors were prioritized for further analysis – CD36, ITGB3, and LRP1 – based on their well-characterized role as membrane receptors and their relevance in SM physiology [37–39]. For each predicted interaction, we sought corroborating evidence through a systematic literature review (Table 1). For ITGB3 and LRP1, only one study per receptor reporting an association with PLIN2 was found. The functional interaction between PLIN2 and ITGB3 appears limited, as both proteins were discussed in the context of PFOA and GenX treatment without direct evidence of physical interaction. LRP1-PLIN2 interaction was supported by experimental evidence of protein colocalization in lipid vacuoles enriched in cholesteryl esters following LRP1-mediated uptake of aggregated LDL in human vascular smooth muscle cells and macrophages [40]. Of particular interest was the CD36-PLIN2 interaction, as it was predicted by PrePPI based on convergent evidence from multiple likelihood ratio calculations spanning structural, phylogenetic, expression, and functional annotation data. This computational prediction was strongly supported by an extensive body of literature co-reporting PLIN2 and CD36 in studies related to lipid metabolism (Table 1). The widespread co-occurrence of these proteins in studies on LD transport and formation suggests a functionally significant interaction in cellular contexts where coordinated regulation of lipid uptake and storage is critical. Moreover, recent evidence indicates that PLIN2 interacts with CD36 to promote colorectal cancer progression via CD36-mediated epithelial–mesenchymal transition [41].

**Table 1:** Identified predicted interactors of PLIN2

PLIN2 predicted interactor	Source of prediction	References
LRP1	IID	[40]
CD36	PrePPI	[41–46]
ITGB3	PrePPI	[47]

Given these data, we analyzed the mRNA expression level of *CD36* in *Vastus lateralis* SM biopsies from HS and MIP. *CD36* transcript levels were significantly higher in HS compared to MIP (Fig. 5B), consistent with previous reports showing higher *CD36* expression in physically active SM [38]. We next investigated whether SM CD36 expression was associated with cPLIN2 levels. Although an inverse correlation was observed, this association did not reach statistical significance (Spearman’s ρ = -0.295, *p* = 0.207), indicating that a direct relationship between these variables cannot be established, likely due to the small size of this cohort. Nevertheless, the combination of bioinformatic prediction, literature evidence, and the differential expression of *CD36* between HS and MIP supports CD36 as a biologically plausible candidate interactor of cPLIN2. However, the proposed cPLIN2-CD36 interaction and its possible role in adipose tissue-skeletal muscle cross-talk remain speculative and further studies are required to confirm this hypothesis and clarify its mechanistic bases.

## Discussion

PLIN2 is one of the most abundant proteins coating LD surfaces. Its role is not limited to stabilization of LD structure, in fact it also participates in fatty acid uptake, LD formation, and lipid storage [1]. PLIN2 is expressed in a wide range of tissues; however, despite its involvement in lipid storage, it is largely absent in mature adipocytes, where PLIN1 and PLIN4 are the predominant LD-associated proteins.

The importance of PLIN2 in human pathology is highlighted by its association with disorders such as insulin resistance, type 2 diabetes, obesity, cardiovascular disease, atherosclerosis, sarcopenia, and fatty liver diseases, as well as by evidence showing that PLIN2 silencing protects against several of these conditions in animal models [1, 48, 49]. However, these protective effects likely result from the absence of PLIN2 in non-adipose tissues (e.g., skeletal muscle, liver), since mature adipocytes normally express low levels of PLIN2, and thus its modulation should not confer additional benefit. Nevertheless, because PLIN2 is expressed during the early phases of adipogenic differentiation [9] and since PLIN2-KO mice are protected from high-fat diet-induced obesity [50], we investigated whether PLIN2 may contribute to adipogenic remodeling. Importantly, the present study was specifically designed to investigate the role of PLIN2 in adipogenic differentiation of DFs. Although DFs are not proper preadipocytes, they represent a biologically informative and mechanistically useful model for studying PLIN2-dependent adipogenic plasticity. Indeed, DFs are capable of undergoing adipogenic differentiation, as previously reported [51, 52], while their less intrinsically adipogenic background allows a more direct evaluation of the specific contribution of PLIN2, with reduced interference from the more complex perilipin network present in committed preadipocytes and mature adipocytes. Notably, preadipocytes express PLIN1 and PLIN4 [53] in addition to PLIN2, making it more difficult to dissect the specific contribution of PLIN2 during adipogenesis. Using DFs cultured in ADM, we have found that the expression of *Plin2* gene is strongly decreased during the early phases of differentiation. These findings suggest that the downregulation of *Plin2* expression may represent a fundamental step in the adipogenic remodeling process. Accordingly, its modulation influences LD growth and expression of adipocyte-related genes, such as *Leptin*, *Adiponectin* and *Fabp4*, suggesting that the reduction of *Plin2* transcript abundance may facilitate adipogenic conversion in this cellular model. This hypothesis is also supported by the fact that the effect on adipogenesis seems to be specific for *Plin2* modulation (and not for *Plin3*, nor for OA treatment). It is known that LD accumulation is a marker of cell senescence [54] and that siPLIN2 induces cell senescence [12]. However, in DFs cultured in ADM we did not observe an increased expression of senescence-associated markers, such as *p21* and *Gdf15*, following siPLIN2 treatment, indicating that the role of PLIN2 may differ across culture conditions, depending on the metabolic and differentiation state of the cells.

Looking for more evidence supporting the idea that PLIN2 is playing an active role in hindering adipogenic differentiation, we have investigated not only the levels of *Plin2* transcripts but also PLIN2 protein. Surprisingly, the protein levels were decreased only by day 3 but not by day 9 of differentiation, when instead an increase was observed upon ADM culture. The discrepancy between the rapid loss of *Plin2* transcript and the persistence of detectable PLIN2 protein may be accounted for by post-transcriptional and post-translational regulatory mechanisms, as previous studies have shown that PLIN2 protein abundance is strongly influenced by LDs association, protein turnover, and the progressive replacement of PLIN2 by PLIN1 during adipocyte maturation [5, 9]. Other studies demonstrated that PLIN2 stability strongly depends on its association with LDs, whereas non-LD-associated PLIN2 undergoes rapid degradation through ubiquitin-proteasome pathways and chaperone-mediated autophagy [55].

Our results are in agreement with a recently published study performed on a different experimental model (OA-induced adipogenic differentiation of muscle satellite cells in cattle [56]) and, most of all, we provided evidence that PLIN2 in cells undergoing differentiation can be extracellularly released (cPLIN2). This fits with previous in vivo data showing an association between plasma cPLIN2 and fat mass [16], suggesting that adipogenic remodeling may contribute, at least in part, to extracellular PLIN2 levels. It is known that PLIN2 takes part in the secretion of LDs in breast milk [6], however, nothing is known so far about PLIN2 in other body fluids and plasma. By similarity with milk, it can be hypothesized that (at least a part of) LDs are secreted by using PLIN2 as an adaptor between intracellular LDs and the inner membrane leaflet [6]. However, since our ExoView analysis indicated that PLIN2^+^ EVs are Tetraspanin-positive (in particular CD63^+^), it is likely that we are looking at extracellular lipidosomes. These are conventional, bilayer-enclosed EVs that have packaged a whole monolayered LD into their lumen during endosomal biogenesis or macroautophagy [57]. We cannot claim at present that PLIN2^+^ EVs account for the whole amount of cPLIN2, and possibly other forms of cPLIN2 exist. Moreover, ELISA measures total detectable extracellular PLIN2 and does not discriminate between EV-associated and non-vesicular forms of the protein. Further studies are needed to clarify this aspect.

We previously reported that cPLIN2 levels are inversely correlated with the amount of muscle mass [16]. Here we report that cPLIN2 levels are higher in physically inactive people with respect to healthy active age-matched controls and are associated with lower muscle strength. It can be thus hypothesized that cPLIN2 is released by preadipocytes and likely endocytosed by active skeletal muscle. Accordingly, we report here that the expression of *CD36*, a lipid transporter that has been indicated as an interactor of PLIN2 bound to plasma and endosomal membranes [58], is lower in *Vastus lateralis* muscle of physically inactive people with respect to active counterparts. The fact that CD36 is known as a fatty acid translocase further suggests that PLIN2^+^ EVs likely carry fatty acids and are therefore derived from (or incorporate) LDs.

This study has also some important limitations. First, although our findings support a role for PLIN2 modulation during DF differentiation, validation in additional adipogenic models will be important in future studies. Second, we were not able at this stage to perform *Plin2* overexpression experiments. Third, we did not extend differentiation beyond 15 days, therefore we do not have data on PLIN2 secretion by mature adipocytes. Finally, the precise mechanisms regulating PLIN2 extracellular release and its potential biological effects remain to be clarified.

## Conclusion

According to our data, the role of PLIN2 during adipogenic differentiation appears to be more complex than previously thought and may go beyond its recognized involvement in LDs stabilization and lipid turnover and metabolism. In particular, our findings suggest that *Plin2* downregulation is a crucial step in the early phases of the adipogenic process and it is needed for correct LDs growth. However, the mechanisms underlying the transition from PLIN2 to PLIN1 during adipocyte maturation remain incompletely understood. The other key observation of this study is the secretion of PLIN2 (cPLIN2) during adipogenic differentiation. Since this phenomenon was observed only upon ADM culture, it is suggested that cPLIN2 release may be a specific feature of adipogenic remodeling. In this context, cPLIN2 may represent an interesting biomarker of adiposity, or even a new player – and, accordingly, a potential therapeutic target – involved in a number of disorders linked to excess adiposity. However, the precise mechanism underlying cPLIN2 release and its biological significance, including the proposed interaction of PLIN2^+^ EVs with skeletal muscle, remain to be clarified and formally proven in future studies.

## Supplementary Information

Below is the link to the electronic supplementary material.

Supplementary figure 1 (PNG 1.28 MB)

High Resolution Image (TIF 24.1 MB)

Supplementary figure 2 (PNG 205 KB)

High Resolution Image (TIF 8.06 MB)

Supplementary figure 3 (PNG 421 KB)

High Resolution Image (TIF 24.1 MB)

Supplementary figure 4 (PNG 659 KB)

High Resolution Image (TIF 24.1 MB)

Supplementary Material 5 (DOCX 15.8 KB)

Supplementary Material 6 (XLSX 17.6 KB)

Supplementary Material 7 (XLSX 12.1 KB)

Conte et al Suppl fig legend

## Data Availability

All data needed to evaluate the conclusions are present in the paper and Supplementary Materials.

## Abbreviations

ADM: Adipogenic differentiation medium
cPLIN2: Circulating PLIN2
DFs: Human primary dermal fibroblasts
DMEM: Dulbecco's Modified Eagle Medium
EVs: Extracellular vesicles
HS: Healthy subjects
LD: Lipid droplet
MIP: Mobility impairment
OA: Oleic acid
PLIN2: Perilipin 2
PrePPI: PREdicted Protein-Protein Interactions database
QS: Quadriceps strength
RNAi: RNA interference
SEM: Scanning Electron Microscopy
SM: Skeletal muscle
TEM: Transmission Electron Microscopy
WAT: White adipose tissue
