# Supplementary material for: Perilipin 2 modulates adipogenic differentiation of primary dermal fibroblasts and is extracellularly released during adipocyte maturation
Source: Cell Mol Life Sci. 2026 Jul 28;83(1):397. doi: 10.1007/s00018-026-06358-z (PMC13710739; doi:10.1007/s00018-026-06358-z)
Supplement: Supplementary file 1 [file 18_2026_6358_MOESM5_ESM.docx]

**Supplementary Table 1. Correlation between circulating PLIN2 (cPLIN2) and clinical parameters.**

**Suppl. TABLE 1.**

| **Correlation with cPLIN2** | **ρ** | **p value** |
| --- | --- | --- |
| BMI | 0.492 | **p<0.001** |
| LEPTIN | 0.843 | **p<0.001** |
| FAT MASS (%)^*^ | 0.815 | **p<0.001** |
| LEAN MASS (%)^*^ | -0.760 | **p<0.001** |
| QS/BMI | -0.406 | **p<0.001** |

**Abbreviations**: QS = quadriceps strength; ρ = Spearman rank correlation coefficients.

**Notes:** Correlations with BMI, LEPTIN, and QS/BMI were calculated using the entire study cohort (HS + MIP). *Correlations with fat mass (%) and lean mass (%) were calculated in HS group only, as body composition measurements were available exclusively for this group. Statistically significant p-values (p < 0.05) are shown in bold.

**Supplementary Table 2. Multiple linear regression analysis of factors associated with plasma cPLIN2 levels**

| **Variable** | **β coefficient** | **SE** | **95% CI** | ***P* value** |
| --- | --- | --- | --- | --- |
| Intercept | 10.38 | 15.05 | -19.43 to 40.19 | 0.498 |
| **Study group (HS vs. MIP)** | **-7.63** | **3.43** | **-14.43 to -0.82** | **0.028** |
| Sex | -2.31 | 3.95 | -10.14 to 5.51 | 0.559 |
| Age | 0.06 | 0.08 | -0.09 to 0.21 | 0.403 |
| BMI | -0.06 | 0.59 | -1.22 to 1.09 | 0.918 |
| **LEPTIN** | **2.43** | **0.22** | **2.00 to 2.87** | **<0.001** |
| RESISTIN | -0.05 | 0.31 | -0.66 to 0.56* | 0.884 |

**Model Statistics**

| Parameter | Value |
| --- | --- |
| R² | 0.70 |
| Adjusted R² | 0.69 |
| Overall model *P* value | <0.001 |

**Notes:** Multiple linear regression analysis was performed with plasma cPLIN2 as the dependent variable and study group (HS *vs* MIP), sex, age, BMI, LEPTIN, and RESISTIN as independent variables. Statistically significant associations (p < 0.05) are shown in bold. Multicollinearity was assessed using the variance inflation factor (VIF); all VIF values were <2.2, indicating no evidence of problematic collinearity.
