# Supplementary material for: Perilipin 2 modulates adipogenic differentiation of primary dermal fibroblasts and is extracellularly released during adipocyte maturation
Source: Cell Mol Life Sci. 2026 Jul 28;83(1):397. doi: 10.1007/s00018-026-06358-z (PMC13710739; doi:10.1007/s00018-026-06358-z)
Supplement: Supplementary file 2 [file 18_2026_6358_MOESM8_ESM.docx]

**SUPPL FIGURE LEGEND**

**SUPPLEMENTARY FIGURE 1**

**Time-course analysis of adipogenic differentiation in human dermal fibroblasts (DFs).** (**A-D**) Light microscopy images of DFs cultured in adipogenic differentiation medium and observed at days 3 (**A**), 6 (**B**), 9 (**C**) and 15 (**D**); lipid droplets become clearly visible from day 6 (black arrows). Magnification 20X. (**E-G, I**) Real-time RT-PCR analysis of DFs cultured in DMEM (control, CTR) and in adipogenic differentiation medium (differentiated cells, DIFF) at days 3, 6, 9, and 15 to assess the level of adipogenic differentiation. (**H**) Western blot analysis of PLIN2, PLIN3, and PLIN1 protein levels in control (CTR) and differentiated (DIFF) cells at day 3 and 9, with adipose tissue (AT) included as a reference. GAPDH was used as a loading control. (**J**) Levels of secreted LEPTIN in the culture supernatant DFs cultured in DMEM (CTR) and in adipogenic differentiation medium (DIFF) at days 3, 6, 9, and 15. Data are expressed as mean ± SD from two independent biological replicates. Statistical analysis was performed using the Kruskal-Wallis test followed by Bonferroni-corrected pairwise comparisons.

**SUPPLEMENTARY FIGURE 2**

**Real-time RT-PCR analysis following *Plin2* or *Plin3* downregulation (siPLIN2 or siPLIN3).** (**A-B**) *Plin2* and *Plin3* transcript levels in DFs cultured in DMEM (control, CTR), DMEM treated with siPLIN2 (siPLIN2 CTR), adipogenic differentiation medium (differentiated cells, DIFF), and pro-adipogenic medium treated with siPLIN2 (siPLIN2 DIFF). (**C-D**) *Plin3* and *Plin2* transcript levels in DFs cultured in DMEM (CTR), DMEM treated with siPLIN3 (siPLIN3 CTR), adipogenic differentiation medium (DIFF), and adipogenic differentiation medium treated with siPLIN3 (siPLIN3 DIFF). (**E-F**) *p21* and *Gdf15* transcript levels in DFs cultured in DMEM (CTR), DMEM treated with siPLIN2 (siPLIN2 CTR), adipogenic differentiation medium (DIFF), and adipogenic differentiation medium treated with siPLIN2 (siPLIN2 DIFF). Data are presented as mean ± SD from two independent biological replicates. Statistical significance was assessed using Student’s *t*-test.

**SUPPLEMENTARY FIGURE 3**

**Real-time RT-PCR analysis following *Plin2* downregulation (siPLIN2) and Brefeldin A and Monensin (B+M) treatment.** (**A-C**) *Plin2*, *Plin1* and *Plin3* transcript levels in DFs cultured in adipogenic differentiation medium (ADM) alone (DIFF), in ADM supplemented with B+M (B+M DIFF), and in ADM following siPLIN2 and B+M treatment (siPLIN2-B+M DIFF). Data are expressed as mean ± SD from two independent biological replicates. For each biological replicate, real-time RT-PCR measurements were performed in technical triplicate and averaged before statistical analysis. Statistical significance was assessed using Student’s t-test.

**SUPPLEMENTARY FIGURE 4**

**Cell viability and PI-based cell death quantification.** **(A–B)** Representative light microscopy images of control (CTR, A) and differentiated (DIFF, B) cells showing comparable cell density, indicating no evident differences in cell viability between the two conditions; magnification: 10×. **(C–D)** Flow cytometry analysis of cell death in CTR (C) and DIFF (D) cells. The percentage of PI-positive (dead) cells is indicated in each panel.
